# *Mycobacterium mageritense* Prosthetic Joint Infection

**DOI:** 10.1155/2020/8845430

**Published:** 2020-07-11

**Authors:** Maria A. Caravedo Martinez, Lucas S. Blanton

**Affiliations:** Department of Internal Medicine, Division of Infectious Diseases, University of Texas Medical Branch, 301 University Boulevard, Galveston, TX 77550-0435, USA

## Abstract

Prosthetic joint infections (PJI) complicate up to 2% of arthroplasties and are usually caused by typical bacterial agents (e.g., staphylococci and streptococci). Although an infrequent cause of PJI, mycobacterial species are difficult to eradicate, as they adhere to hardware, form biofilms, and have high rates of antimicrobial resistance. *Mycobacterium mageritense* is a rapidly growing *Mycobacterium* that has been infrequently described as a cause of surgical and device-related infections. We herein described a case of prosthetic knee infection due to *M. mageritense*. The patient was treated with removal of hardware, antimicrobials, and prosthetic knee reimplantation with a good outcome. To our knowledge, *M. mageritense* has not been previously described as a cause of PJI in the medical literature.

## 1. Introduction

In the last several decades, there has been a great increase in the number of performed joint arthroplasties, with subsequent improvement in mobility and quality of life in recipients of these devices. Unfortunately, prosthetic joint infections (PJI) complicate up to 2% of arthroplasties [1]. These infections are usually caused by typical bacterial agents, such as staphylococci, streptococci, and those in the family *Enterobacteriaceae* [2]. Although mycobacterial species are infrequent causes of PJI, when found to be the causative agent, they are difficult to manage. Factors that contribute to these challenging infections include the ability of the bacteria to adhere to hardware, form biofilms, and their often intrinsic resistance to antibiotics [3]. *Mycobacterium mageritense* is a rapidly growing nontuberculosis mycobacteria that was first isolated in Spain in 1987 and described as a unique species in 1997 by Domenech and colleagues [4]. It was later described as a culprit of disease in the United States in 2002 [5]. Cardiovascular device infections, catheter-related infections, surgical wound infections, pulmonary infections, and osteomyelitis related to this *Mycobacterium* have been described [6–10]. To our knowledge, there have been no cases of PJI related to this organism documented in the medical literature. Here, we present a case of prosthetic joint infection related to *M. mageritense*.

## 2. Case

**Figure 1:**
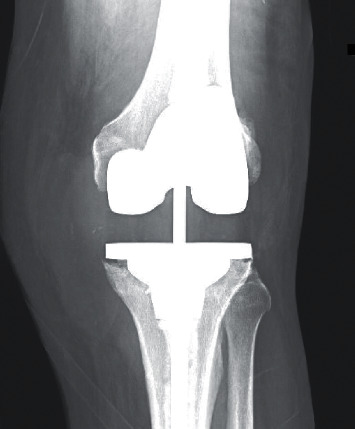
X-ray of the left knee demonstrating perihardware lucencies at the level of the tibial trays.

A 66-year-old male with hypertension, hyperlipidemia, gastroesophageal reflux disease, and a history of bilateral total knee arthroplasty (right in 2009 and left in 2015) was in his usual state of health, until experiencing left knee pain after stumbling down some stairs and having the sensation of twisting his left knee in July 2016. He had revision of the left knee later that month due to the appearance of hardware loosening on x-ray. The surgery was without complications. Aerobic, anaerobic, fungal, and mycobacterial cultures all returned negative. However, 1 week after surgery, he developed wound dehiscence and worsening pain. He presented to the emergency department and was taken for irrigation and debridement of the left knee with insert exchange of the left total knee arthroplasty; he was given vancomycin and piperacillin-tazobactam empirically. Cultures remained negative, albeit only aerobic and anaerobic tissue cultures were sent, and the patient was discharged on 6 weeks of empiric ceftriaxone and was then transitioned to oral doxycycline. In January 2017, he developed worsening swelling and pain. An aspiration of a fluid-filled collection medial to the distal portion of the prior surgical incision was performed. Approximately, 15 ml of purulent appearing fluid was removed, and analysis revealed a white cell count of 30,250 cells/*µ*l with neutrophil predominance (86%). Aerobic, anaerobic, and fungal cultures revealed no growth, but culture for acid-fast bacilli grew *M. mageritense.* The patient was referred to the infectious disease clinic. At that time, the patient denied systemic symptoms of infection (e.g., fever, chills, or night sweats). Physical exam was significant for mild infrapatellar swelling, but no overlying erythema or drainage was noted. Labs showed an ESR of 82 mm/hour and CRP of 13.4 mg/dL. While awaiting antimicrobial susceptibilities, the patient was started on empiric treatment with ciprofloxacin 500 mg and trimethoprim-sulfamethoxazole (TMP-SMX) 160–800 mg, both twice daily. The isolate was eventually found to be susceptible to numerous agents (Table 1). At that time, his surgical team believed that the infection involved only the soft tissues and deferred additional surgical manipulation. The patient continued oral antibiotics, and on follow-up in March 2017, he was noted to have new changes on x-ray of his left knee. The films demonstrated soft tissue swelling and perihardware lucencies at the level of the tibial trays (Figure 1). With radiographic evidence to support prosthesis-related infection, the patient underwent explantation of the hardware with spacer placement. After explant, he was initiated on amikacin 750 mg intravenous every 12 hours and imipenem 1 g intravenous every 8 hours for 2 months. Periprosthetic mycobacterial tissue cultures taken at the time of explant revealed no growth. Two months after explant, he underwent reimplantation of hardware and was transitioned to oral antibiotics with ciprofloxacin and TMP-SMX. Approximately three months after reimplantation, as the patient was becoming more physically active, he again developed swelling and pain. Aspiration at that time yielded normal appearing fluid, which failed to grow any organisms. Subsequently, these symptoms improved. The patient was maintained on oral antibiotics for a year. He continues to do well on follow-up and is without signs or symptoms of infection.

## 3. Discussion

*Mycobacterium mageritense* is a rapidly growing nontuberculous *Mycobacterium*. The rapid growers include numerous mycobacterial species grouped into 6 taxonomic groups [11]. *Mycobacterium chelonae*, *M. abscessus*, and *M. fortuitum* are the most frequently isolated species and are known culprits of hardware infection [12]. Rapidly growing mycobacteria (RGM) often exhibit growth within seven days, and although a variety of phenotypic features have been used to aid in their speciation, molecular techniques or use of matrix-assisted laser desorption ionization-time of flight mass spectrometry (MALDI-TOF) have been more recently used to accurately differentiate between the many closely related species. RGM are ubiquitously found in the environment. Infection can follow contamination of wounds with soil, has been described in association with contaminated footbaths at nail salons, has been linked to therapeutic tumor necrosis factor alpha inhibition, and occur in nosocomial outbreaks [11]. Our patient had no obvious exposure or inciting event to explain his infection. He had no immunocompromising condition and was on no immunosuppressing medications. A source of traumatic inoculation was never recognized, nor was *M. mageritense* recognized as causing clusters of infection at our institution. It is hypothesized that an inadvertent and unrecognized inoculation of this organism into the surgical wound caused the resulting infection.

Considering the relatively infrequent occurrence of RGM associated PJI, ideal management is unknown, as controlled trials to evaluate various treatments are not available. However, observational data support that removal of the infected foreign body in addition to antibiotics is critical [12]. In the aforementioned case report, the initial lack of improvement while on ciprofloxacin and TMP-SMX was likely related to retention of the hardware. Although cultures were negative at the time of explantation of the infected joint, previous antimicrobial use may have inhibited growth of *M. mageritense* at that time. The use of multiple active antimicrobials is recommended to avoid the emergence of resistance while on a single agent. For example, there is a risk of developing mutational resistance with fluoroquinolone monotherapy [5]. Other drugs of therapeutic potential, as demonstrated *in vitro* and as documented in case reports, include imipenem, cefoxitin, sulfonamides, linezolid, and fluoroquinolones. A study performed by Wallace and colleagues on 11 isolates of *M. mageritense* showed susceptibility to ciprofloxacin and TMP-SMX [5]. All isolates were susceptible or intermediate in susceptibility to amikacin, linezolid, imipenem, and cefoxitin; they were intermediate or resistant to tobramycin. All 11 isolates were resistant to clarithromycin.

To our knowledge, this is the first report of *M. mageritense* causing PJI. There are no controlled studies to guide the optimal duration of parenteral and oral antibiotics for *M. mageritense* in the setting of PJI, but as in the case of other RGM, hardware removal and prolonged antimicrobial therapy are key [12]. Our patient did well with explant, 2 months of intravenous therapy, reimplantation, and transition to an oral regimen for a year-long course. He remained free of complications in the year of follow-up monitoring after his treatment course.

## Figures and Tables

**Table 1:** The *Mycobacterium mageritense* isolate demonstrating susceptibility to several antimicrobial agents.

Antibiotic	MIC (*µ*g/ml)	Interpretation
Amikacin	8	Susceptible
Cefoxitin	32	Intermediate
Ciprofloxacin	0.5	Susceptible
Clarithromycin	≥32	Resistant
Doxycycline	2	Intermediate
Imipenem	4	Susceptible
Linezolid	≤1	Susceptible
Minocycline	≤1	Susceptible
Moxifloxacin	≤0.25	Susceptible
Trimethoprim-sulfamethoxazole	1/19	Susceptible

MIC = minimum inhibitory concentration.

## Data Availability

The clinical data used to support the findings of this study are included within the article.
